# Intensive Multidisciplinary Intervention for Young Children With ARFID: Clinical Outcomes and Parental Experiences From a Prospective Cohort Study

**DOI:** 10.1002/eat.70030

**Published:** 2026-01-16

**Authors:** Helena Holmäng, Katarzyna Brimo, Anna Ås, Lisa Dinkler, Rachel Bryant‐Waugh, Christopher Gillberg, Maria Råstam, Maj‐Britt Posserud

**Affiliations:** ^1^ Gillberg Neuropsychiatry Centre Institute of Neuroscience and Physiology Gothenburg Sweden; ^2^ Barnspecialistmottagningen Munin Uppsala Sweden; ^3^ Department of Medical Epidemiology and Biostatistics Karolinska Institutet Stockholm Sweden; ^4^ Maudsley Centre for Child and Adolescent Eating Disorders, South London and Maudsley NHS Foundation Trust London UK; ^5^ Department of Child and Adolescent Psychiatry, Institute of Psychiatry, Psychology and Neuroscience King's College London London UK; ^6^ Department of Clinical Sciences Lund Lund University Lund Sweden; ^7^ University of Bergen Bergen Norway

**Keywords:** ARFID, caregiver involvement, clinical outcomes, intensive multidisciplinary intervention, parental experience, sensory‐based intervention, treatment satisfaction, young children

## Abstract

**Objective:**

To assess clinical outcomes and parental experiences following an intensive multidisciplinary intervention (IMI) for children with Avoidant Restrictive Food Intake Disorder (ARFID).

**Method:**

A prospective cohort of 28 children (aged 2–8 years) with severe eating difficulties, all meeting ARFID diagnostic criteria at baseline, participated in a 3‐day assessment and an 8‐day IMI involving guided mealtimes, play‐based exposures, nutritional counseling, and caregiver coaching. Clinical outcomes were assessed using the Pica, ARFID, and Rumination Disorder Interview (PARDI) at baseline and at 7–15 months post‐intervention. Parental experience was evaluated using the Experience of Service Questionnaire (ESQ) and the study‐specific Eating Intervention Experience Questionnaire (EIEQ).

**Results:**

At follow‐up, 27 of 28 children continued to meet ARFID criteria; however, overall symptom severity decreased significantly (*M* = 3.55 → 2.57, *d* = 0.80, *p* < 0.001), with reductions in both the sensory (*M* = 2.77 → 2.21, *d* = 0.63, *p* = 0.002) and low‐interest profiles (*M* = 3.48 → 2.50, *d* = 0.93, *p* < 0.001), alongside improvements in nutritional status and growth‐related criteria. Parents reported high satisfaction with the intervention, valuing the multidisciplinary approach, tailored support, and practical strategies.

**Discussion:**

Findings suggest that intensive, multidisciplinary, nondirective interventions may yield clinically meaningful benefits for young children with severe ARFID, particularly those with combined sensory sensitivity and low interest in eating profiles. Although full remission was uncommon within the follow‐up period, the intervention supported symptom reduction and caregiver competence. Larger controlled studies are needed to establish efficacy and guide development of sustainable models of care.

## Summary

- We evaluated an 8‐day intensive multidisciplinary intervention for young children with ARFID, using validated diagnostic measures and parent‐reported experiences.
- Children showed meaningful reductions in ARFID symptom severity and parents reported high satisfaction with the intervention.
- Findings suggest that nondirective, caregiver‐focused interventions may benefit young children with severe, long‐standing eating difficulties, particularly those presenting with sensory sensitivity and low interest in eating.

## 1 Background

Avoidant Restrictive Food Intake Disorder (ARFID) is a feeding and eating disorder characterized by severely limited food intake, leading to weight loss or faltering growth in children, nutritional deficiencies, reliance on oral supplements or tube feeding, and/or significant psychosocial impairment (American Psychiatric Association 2013; World Health Organization 2021). Unlike other eating disorders, ARFID is not primarily motivated by concerns about weight or shape. Instead, food avoidance is typically driven by one or more of the following: (i) low interest in eating or food, (ii) fear of aversive consequences (e.g., choking or vomiting), (iii) and/or sensory aversions to food characteristics (e.g., smell, taste, or texture) (American Psychiatric Association 2013). As such, ARFID presents heterogeneously, with wide variation in clinical presentation.

ARFID can develop at any age but frequently presents in childhood and adolescence (Sanchez‐Cerezo et al. 2023). Early detection and intervention are therefore critical to preventing the progression of eating difficulties and mitigating adverse physical and psychosocial outcomes later in life (James et al. 2024; Silvers and Erlich 2023). Moreover, children with ARFID frequently present with co‐occurring physical and neurodevelopmental concerns, which may further complicate diagnosis and treatment (Sanchez‐Cerezo et al. 2023; Wronski et al. 2025).

Despite the importance of early intervention, formal treatment guidelines for young children with ARFID have yet to be established (American Psychiatric Association 2023; National Institute for Health and Care Excellence 2020; Socialstyrelsen 2024). International consensus emphasizes a multifaceted approach that includes parent involvement, developmentally appropriate strategies, and flexibility to accommodate individual needs (Archibald and Bryant‐Waugh 2023; Kambanis and Thomas 2023; Perez and Pesce 2025). Although the evidence base remains limited, intensive multidisciplinary interventions (IMIs) and lower‐intensity, parent‐mediated approaches have emerged as promising options for young children with ARFID (Kambanis and Thomas 2023; Willmott et al. 2024). Interventions in both formats commonly emphasize caregiver training and the use of strategies grounded in behavioral frameworks (Willmott et al. 2024). However, IMIs represent a higher‐intensity model, typically delivered in inpatient or day‐treatment settings where a multidisciplinary team (e.g., physician, psychologist, dietitian, speech and language therapist, special needs educator) supports children and families across multiple daily mealtimes over consecutive days or weeks. This model is particularly indicated for long‐standing, severe eating difficulties, especially when lower‐intensity alternatives have proven insufficient or comorbidities complicate treatment (Kambanis and Thomas 2023; Perez and Pesce 2025; Sharp et al. 2017; Volkert et al. 2021).

Within the broader IMI literature for pediatric ARFID, there is emerging support for approaches grounded in behavioral, family‐based and cognitive‐behavioral frameworks (Billman et al. 2022; Bryson et al. 2018; Dolman et al. 2021; Dumont et al. 2019; Hellner et al. 2025; Lane‐Loney et al. 2022; Lenz et al. 2018; Reilly et al. 2020; Sharp et al. 2016, 2020; Volkert et al. 2021). For younger children, the field largely describes behavioral interventions delivered in US‐based programs (e.g., Sharp et al. 2016, 2020, 2024; Volkert et al. 2021). These often involve structured mealtime exposures with therapist‐ or caregiver‐led prompting and reinforcement techniques (e.g., contingent and differential reinforcement), targeting specific goals related to eating behavior and nutritional adequacy. Although findings are promising, the IMI evidence base remains constrained by few prospective studies, limited use of validated diagnostic instruments, and inconsistent outcome measures (Willmott et al. 2024). For younger children, evidence is further limited by the predominance of IMIs grounded in behavioral frameworks, with little evaluation of alternative models in non‐US settings.

The present study prospectively evaluates an IMI for young children with ARFID, grounded in a nondirective model, examining clinical outcomes and parental experiences.

## 2 Method

### 2.1 Study Setting and Procedure

This prospective cohort study was conducted at the Folke Bernadotte Regional Habilitation Centre (FBHC) in Uppsala, Sweden. The intervention was delivered between January 2022 and February 2023, during which FBHC operated a national specialist unit providing multidisciplinary assessment and treatment for children with complex feeding and eating difficulties. The unit closed in 2023 due to organizational restructuring, preventing further scheduling.

Upon referral, families completed a 3‐day assessment at FBHC, which also introduced the principles of the intervention. All families who completed the assessment remained interested and were offered an 8‐day intervention approximately 6 months later, allowing time for coordination and family preparation.

Data were collected between January 2022 and April 2024 using validated diagnostic interviews to assess clinical outcomes and caregiver questionnaires capturing both quantitative and qualitative parental experiences. The Pica, ARFID, and Rumination Disorder Interview (PARDI) was conducted prior to or at the start of the 8‐day intervention (baseline) and again at follow‐up, either in person, by phone, or via video call. On the final day of the intervention, parents completed the Experience of Service Questionnaire (ESQ) and the study‐specific Eating Intervention Experience Questionnaire (EIEQ) on‐site and returned them in sealed envelopes to ensure confidentiality.

Participation was invited during the intake session of the 3‐day phase, or—if the initial visit had occurred before study onset—at the start of the 8‐day intervention period. The study was approved by the Swedish Ethical Review Authority (reference numbers: 2020‐01284, 2020‐03908), and written informed consent was obtained from all participating parents.

### 2.2 Participants

All families who received intervention at FBHC between January 2022 and February 2023 and provided informed consent were included. No additional inclusion or exclusion criteria were applied beyond FBHC's standard referral and acceptance procedures. Referral eligibility required prior assessment and initial management by a pediatrician, dietitian, and speech and language therapist in the child's home region. Children were eligible for referral if they presented with severe feeding and eating difficulties that could not be adequately managed within local services, were under 7 years of age at the time of referral, and were medically stable (i.e., not requiring inpatient care).

**FIGURE 1:**
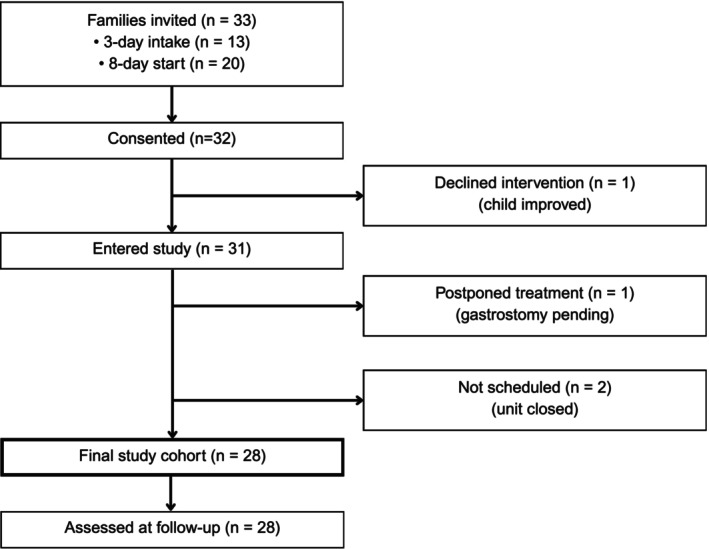
Study enrolment flow. Flow of invited families, consent, exclusions, final cohort and follow‐up participation.

A total of 33 families were invited to participate in the study, either during the intake session of the 3‐day assessment phase (*n* = 13), or—if they had completed this phase prior to the study's onset—at the start of the 8‐day intervention period (*n* = 20). Of these, 32 provided consent. One family declined to proceed with the 8‐day intervention, reporting that the child's feeding had sufficiently improved. In one case, treatment was postponed due to pending medical procedures (gastrostomy placement), and two children could not be scheduled before the unit closed in 2023. The final study cohort thus comprised 28 children (aged 24–96 months) and their families, all of whom completed the follow‐up assessment (see Figure 1).

### 2.3 Intervention

The FBHC intervention is a locally developed model that builds on decades of clinical experience with children presenting with severe eating difficulties. It is grounded in a holistic perspective emphasizing a comprehensive understanding of each child's needs and family context.

The intervention's underlying rationale is that, although children with severe eating problems may have diverse medical or developmental histories, they often share pronounced sensory sensitivities and limited engagement in eating, sometimes accompanied by oral‐motor limitations. Pressuring the child to eat often exacerbates avoidance and stress, reinforcing maladaptive mealtime patterns. The intervention is directed toward interrupting these cycles, reducing mealtime burden, and promoting positive, developmentally supportive eating experiences while strengthening parental capacity to support their child's eating development.

In the initial 3‐day visit, the team conducted a structured assessment including observed mealtimes, parental interviews, play‐based sessions, and nutritional counseling while introducing the guiding principles of the intervention and establishing a foundation for continued work. Preparatory information, collected in advance through a 3‐day food diary and communication with the child's preschool and local healthcare providers, further informed the assessment.

The subsequent intervention period followed a structured schedule, with individual adaptations to meet each family's needs. In all but two of the 28 cases, both parents were present and actively involved throughout. Each day, families participated in one clinician‐guided mealtime, one to two independent practice meals, and one play or activity session. During the intervention period, families also attended three to five dietetic consultations. Parent sessions were conducted without the child present, while a childcare assistant provided age‐appropriate activities.

The intervention was delivered by a multidisciplinary team comprising a speech and language therapist, special needs educator, and dietitian, supported by a childcare assistant. The team shared responsibility for assessment and intervention delivery, with joint intake and history‐taking forming a foundation for collaboration with parents and promoting a shared understanding of the child's needs.

The speech and language therapist applied a nondirective approach based on the team's own model, *Guidance During Meals*, emphasizing low‐pressure and responsive interactions (Ek et al. 2016). Key principles included offering food without pressure, avoiding direct questions or praise related to eating, and using descriptive language to support sensory exploration (see Supporting Information Summary 1 for applied strategies and examples). To further support the child's gradual engagement with food, a sensory‐based progression was used, drawing on the *Steps to Eating* hierarchy (Toomey 2010), adapted into the *Guidance During Meals* model with step‐specific examples and visual supports. Adults were encouraged to model curiosity, describe sensory qualities, and explore food together with the child—without expectation. Meals were video‐recorded and reviewed with parents to reflect on interaction strategies. Parents alternated between guiding and observing the meal, supported by individual feedback and brief written prompts for independent practice.

Building on the same principles, the special needs educator led individualized play‐based sessions designed to promote food‐related curiosity, sensory tolerance, and oral‐motor development. Activities such as pretend play, baking, and sensory games were tailored to each child and often used to introduce new foods in a non‐pressuring context. Parents were gradually supported to take a more active role—initially observing, then practicing the interaction style themselves—mirroring the approach used during mealtimes.

The dietitian conducted a detailed assessment of the child's nutritional status, feeding history, and gastrointestinal health. Nutritional intake was optimized through individualized adjustments involving food, supplements, or tube feeding when necessary. Meal plans prioritized foods that matched the child's sensory and motor abilities, emphasizing reduced performance pressure and reframed expectations. Accepted products were first introduced in play and then, if tolerated, incorporated into meals.

Intervention fidelity was supported through shared team routines, structured schedules, and video‐recorded mealtimes used for reflective discussions with parents and internal team review. Direct coaching, written prompts, and repeated modeling ensured consistency in interaction strategies across sessions. While individual adaptations were made to meet each family's needs, these remained within the framework of the core intervention principles. The intervention was thus delivered as intended in all essential components.

### 2.4 Measures

#### 2.4.1 PARDI

The PARDI is a semi‐structured interview designed to diagnose ARFID according to DSM‐5 criteria, and to provide an overall severity score as well as profile scores across three domains of ARFID: sensory sensitivity, lack of interest in food or eating, and concern about aversive consequences (Bryant‐Waugh et al. 2019). The interview includes both yes/no questions and Likert‐scale items rated from 0 to 6, where 6 indicates the highest level of symptom severity. These ratings inform the profile and overall severity scores.

The PARDI can be administered by professionals from various backgrounds; however, training is essential to ensure consistent interpretation of items and appropriate question delivery. In this study, interviews were conducted by a member of the FBHC team (A.Å.), who received 1 day of training delivered by Rachel Bryant‐Waugh, one of the developers of the instrument.

The parent/carer‐report version of the PARDI was used as the outcome measure to assess clinical features and changes from baseline to follow‐up, including diagnostic status, ARFID severity scores, profile scores, and the number of items rated 4 or higher within each profile. Due to the young age of the children (24–96 months at baseline), items assessing weight and shape concerns—used to exclude other eating disorders—were not included. The health checklist included in the PARDI was used to contextualize eating behaviors and identify potential exclusion criteria for an ARFID diagnosis.

#### 2.4.2 ESQ

The ESQ is a brief, standardized measure used to assess experiences of child and adolescent mental health services (Attride‐Stirling 2002). The questionnaire includes 12 items, each rated on a 3‐point Likert scale: “Certainly True,” “Partly True,” or “Not True,” with an additional “Don't Know” option. It also includes three free‐text sections inviting respondents to comment on what was good about the care, what could be improved, and whether they had anything else to share about their experience. In the present study, the parent/carer version of the ESQ was administered.

#### 2.4.3 EIEQ

In addition to the ESQ, parents completed the EIEQ, an 11‐item questionnaire developed by our research team to assess parental experiences with aspects of the intervention not addressed by the ESQ, including intervention coherence, the value of multidisciplinary input, parental learning and support, and observed changes in the child's eating behavior. Each item was rated on a 7‐point Likert scale ranging from 1 (“Not True”) to 7 (“Certainly True”), with an optional 0 (“Not Applicable”) response.

In Section 3, responses from the EIEQ are presented using the following categorization: 7 = “Certainly true,” 5–6 = “Partly true,” 4 = “Neutral,” 1–3 = “Not true,” and 0 = “Not applicable.” The EIEQ itself, along with the full distribution of responses (n/%) across all response options (0–7), is provided in the Supporting Information (Form 1 and Table S1, respectively).

### 2.5 Data Analysis

Quantitative analyses were performed in IBM SPSS Statistics 29. Descriptive statistics were used to summarize demographics and outcomes. Paired‐sample *t* tests assessed pre‐ to post‐intervention changes in PARDI severity and profile scores, anthropometric measures, and the mean number of items rated ≥ 4 within each profile. McNemar's tests were used to examine changes in the proportion of children meeting each ARFID diagnostic criterion. Statistical significance was set at *p* < 0.05. ESQ and EIEQ responses were reported descriptively, including the “Don't know” and “Not applicable” categories.

Qualitative data from ESQ free‐text responses were analyzed using Braun and Clarke's (2006) six‐phase thematic analysis. Codes were generated inductively, grouped into candidate themes, and refined to ensure internal coherence and clear distinctions. Representative quotations illustrate each final theme.

## 3 Results

### 3.1 Baseline Characteristics

Twenty‐eight children (15 boys, 13 girls) aged 24–96 months (*M* = 54, SD = 19) were enrolled. Most children were within the normal BMI range at baseline (64%), while 25% were classified as underweight and 11% as overweight or with severe obesity (see Table 1). Parent‐reported medical and developmental concerns were common, with 24 children (86%) presenting with at least one (see Table 2). Although some of these could plausibly have affected eating—including gastroenterological problems (68%) and food allergies (36%)—the severity of their eating disturbances was considered disproportionate, consistent with ARFID diagnostic criteria.

**TABLE 1:** BMI categories based on the International Obesity Task Force (IOTF) classification.

IOTF BMI category	Baseline (*n* = 28)	Follow‐up (*n* = 27)
Severe thinness (−3)	1 (3.6%)	0 (0.0%)
Moderate thinness (−2)	1 (3.6%)	2 (7.4%)
Mild thinness (−1)	5 (17.9%)	2 (7.4%)
Normal weight (0)	18 (64.3%)	22 (81.5%)
Overweight (1)	2 (7.1%)	1 (3.7%)
Obesity (2)	0 (0.0%)	0 (0.0%)
Severe obesity (3)	1 (3.6%)	0 (0.0%)

*Note*: One child's anthropometric data were unavailable at follow‐up (*n* = 27); this child was in the *Normal weight* category (0) at baseline. Anthropometric data were obtained using the Pica, ARFID, and Rumination Disorder Interview (PARDI). BMI categories follow IOTF classification. Terminology reflects IOTF category labels; people‐first language is used in the text.

Abbreviations: BMI = body mass index, IOTF = International Obesity Task Force.

**TABLE 2:** Parent‐reported medical and developmental concerns at baseline, assessed with the Physical and Mental Health Checklist in the Pica, ARFID, and Rumination Disorder Interview (PARDI).

Medical/developmental concern	*n* (%)
Gastroenterological problems	19 (67.9%)
Respiratory problems	12 (42.9%)
Birth‐related medical problems	12 (42.9%)
Food allergies	10 (35.7%)
Structural abnormalities	8 (28.6%)
Cardiac problems	6 (21.4%)
Neurological problems	4 (21.4%)
*Other medical conditions*	
Coeliac disease	1 (3.6%)
Lymphadenopathy	1 (3.6%)
Primary immunodeficiency	1 (3.6%)
Autism spectrum disorder	2 (7.1%)
No medical or developmental concerns	4 (14.2%)

*Note: n* = 28. Children may be represented in more than one category.

At baseline, all children met diagnostic criteria for ARFID. Criterion A1 (weight loss/faltering growth) was met by 39% of children, and Criterion A2 (nutritional deficiency) by 18%. Criterion A3 (dependence on supplements/tube feeding) was common (82%), with 11 children receiving oral supplements (nutritional drinks or pudding), 11 tube feeding, and one child receiving a vitamin supplement; however, all children also obtained part of their energy requirements from solid food. Criterion A4 (psychosocial impairment) was universal (100%). The mean ARFID severity score was 3.55 (SD = 0.88), with higher scores in the sensory (*M* = 2.77, SD = 1.01) and interest profiles (*M* = 3.48, SD = 1.04) compared to the concern profile (*M* = 0.07, SD = 0.17). All children showed elevations in both the sensory and interest profiles, and most had multiple items rated ≥ 4 in these, indicating a combined presentation (see Table 3).

**TABLE 3:** Descriptive and inferential statistics for ARFID diagnosis, severity scores, profile scores, and item ratings at baseline and follow‐up (PARDI).

Measure	Baseline (*M* ± SD or *n*, %)	Follow‐up (*M* ± SD or *n*, %)	Test statistic	*p*	Cohen's *d*
ARFID diagnosis met	28 (100%)	27 (92.9%)	*χ* ^*2*^(1) = 0.00	1.000	—
Criterion A1 met	11 (39.3%)	4 (14.3%)	*χ* ^*2*^(1) = 4.20	0.039	—
Criterion A2 met	5 (17.9%)	2 (7.1%)	*χ* ^2^(1) = 0.80	0.375	—
Criterion A3 met	23 (82.1%)	23 (82.1%)	*χ* ^2^(1) = 0.50	0.500	—
Criterion A4 met	28 (100%)	26 (92.9%)	*χ* ^2^(1) = 0.50	0.500	—
ARFID severity score	3.55 ± 0.88	2.57 ± 0.94	*t*(27) = 4.24	< 0.001	0.80
Sensory profile score	2.77 ± 1.01	2.21 ± 1.01	*t*(27) = 3.35	0.002	0.63
Interest profile score	3.48 ± 1.04	2.50 ± 0.94	*t*(27) = 4.91	< 0.001	0.93
Concern profile score	0.07 ± 0.17	0.10 ± 0.38	*t*(27) = −0.41	0.682	−0.08
Items rated ≥ 4 (sensory)	4.86 ± 2.45	2.89 ± 2.59	*t*(27) = 4.28	< 0.001	0.81
Items rated ≥ 4 (interest)	6.75 ± 2.78	3.54 ± 3.01	*t*(27) = 4.90	< 0.001	0.93
Items rated ≥ 4 (concern)	0 ± 0	0.15 ± 0.77	*t*(27) = −1.00	0.327	−0.19

*Note*: Higher scores indicate greater symptom severity. Criterion A1 reflects significant weight loss, failure to achieve expected weight gain, or flatering growth and does not require the child to be underweight; Table 1 reports BMI categories based on the IOTF classification at measurement.

Abbreviations: ARFID = Avoidant Restrictive Food Intake Disorder, *d* = Cohen's *d*, df = degrees of freedom, *M* = mean, *p* = probability value, PARDI = Pica, ARFID, and Rumination Disorder Interview, SD = standard deviation, *t* = paired‐samples *t* test statistic, *χ* ^2^ = McNemar's test statistic.

### 3.2 Clinical Outcomes at Follow‐Up

Follow‐up interviews occurred 7–15 months posttreatment (*M* = 13, SD = 2), at which point children were aged 45–112 months (*M* = 70, SD = 18). The number of children within the normal BMI range increased to 22 (82%). The proportion classified as underweight decreased to 4 (15%), and only one (4%) child was overweight. No children remained in the severe thinness or severe obesity categories. Despite these shifts in BMI category distribution, no significant changes were observed in continuous anthropometric measures between baseline and follow‐up (see Tables 1 and 4).

**TABLE 4:** Baseline and follow‐up anthropometric measures with paired‐samples *t* tests and effect sizes.

Measure/category	Baseline (*M* ± SD)	Follow‐up (*M* ± SD)	*t* (df)	*p*	Cohen's *d*
Height (SDS)	−1.02 ± 1.75	−0.94 ± 1.78	−0.40 (26)	0.693	−0.08
Weight (SDS)	−0.82 ± 1.35	−0.80 ± 1.19	−0.15 (26)	0.885	−0.03
BMI (SDS)	−0.29 ± 1.37	−0.47 ± 1.08	0.83 (26)	0.412	0.16

*Note*: One child's anthropometric data were unavailable at follow‐up (*n* = 27). Anthropometric measures are expressed as SDS (standard deviation score) and were obtained using the Pica, ARFID, and Rumination Disorder Interview (PARDI).

Abbreviations: *d* = Cohen's *d*, df = degrees of freedom, *M* = mean, *p* = probability value, SD = standard deviation, *t* = paired‐samples *t* test statistic.

At follow‐up, 27 of 28 children continued to meet ARFID diagnostic criteria. Criteria A1 (14%) and A2 (7%) were met less frequently, and Criterion A3 continued to be met by 82% of children; however, one child had transitioned from tube feeding to oral nutritional supplements. On the corresponding items (proportion of daily energy from supplements/tube feeds), mean scores decreased from 3.46 to 2.96 for oral supplements and from 2.21 to 1.96 for tube feeding (*d* = 0.45 and 0.47, respectively; both *p* < 0.05). Criterion A4 remained highly prevalent (93%). ARFID severity scores significantly decreased from 3.55 to 2.57 (*d* = 0.80), with reductions observed in both the sensory (*d* = 0.63) and interest profiles (*d* = 0.93). The number of children with items rated ≥ 4 also declined in both profiles, while concern profile scores remained negligible (see Table 3). In exploratory analyses, neither changes in severity scores nor in sensory and interest profile scores were significantly associated with age or sex.

### 3.3 Parental Experiences

All parents completed the ESQ, and 25 (89%) also completed the EIEQ, with two missing responses for items 10–11.

Parents reported high overall satisfaction with the care received, particularly regarding relational aspects: 100% responded “Certainly True” to feeling treated well, having their views taken seriously, and perceiving collaboration among professionals. Ratings for practical aspects were more varied, with 71% selecting the highest rating for the comfort of the facilities, and 57% for the timing of treatment in relation to other commitments (see Table 5).

**TABLE 5:** ESQ item response distribution (*n*, %).

Item	Certainly true	Partly true	Not true	Don't know
I feel that the people who have seen my child listened to me	27 (96.4%)	1 (3.6%)	0 (0.0%)	0 (0.0%)
2 It was easy to talk to the people who have seen my child	27 (96.4%)	1 (3.6%)	0 (0.0%)	0 (0.0%)
3 I was treated well by the people who have seen my child	28 (100.0%)	0 (0.0%)	0 (0.0%)	0 (0.0%)
4 My views and worries were taken seriously	28 (100.0%)	0 (0.0%)	0 (0.0%)	0 (0.0%)
5 I feel the people here know how to help with the problem I came for	24 (85.7%)	3 (10.7%)	1 (3.6%)	0 (0.0%)
6 I have been given enough explanation about the help available here	24 (85.7%)	2 (7.1%)	0 (0.0%)	2 (7.1%)
7 I feel that the people who have seen my child are working together to help with the problem(s)	28 (100.0%)	0 (0.0%)	0 (0.0%)	0 (0.0%)
8 The facilities here are comfortable (e.g., waiting area)	20 (71.4%)	8 (28.6%)	0 (0.0%)	0 (0.0%)
9 The appointments are usually at a convenient time (e.g., don't interfere with work, school)	16 (57.1%)	6 (21.4%)	4 (14.3%)	2 (7.1%)
10 It is quite easy to get to the place where the appointments are	27 (96.4%)	1 (3.6%)	0 (0.0%)	0 (0.0%)
11 If a friend needed similar help, I would recommend that he or she come here	27 (96.4%)	1 (3.6%)	0 (0.0%)	0 (0.0%)
12 Overall, the help I have received here is good	27 (96.4%)	1 (3.6%)	0 (0.0%)	0 (0.0%)

*Note*: Responses were rated on a 3‐point scale (“Certainly true”, “Partly true,” “Not true”), with an additional “Don't know” option.

Abbreviation: ESQ = Experience of Service Questionnaire.

Responses to the EIEQ indicated positive parental experiences with the intervention. Across most items, nearly all parents rated their experience as certainly true or partly true. These included the therapeutic approach and communication style (96%), continuity of care (100%), and involvement of a multidisciplinary team (100%). Similarly, high agreement was seen for items on caregiver discussions, sensory understanding, and receiving nutritional guidance.

Greater variability was observed regarding items assessing perceived changes in the child's eating behavior. For example, 75% of parents provided upper‐range ratings for increased interest in eating, while 21% rated this item in the lower range. Likewise, while 71% of parents gave positive ratings for observed improvements in eating, the remaining responses fell into the “Neutral,” “Not true,” or “Not applicable” categories, indicating a broader range of responses regarding short‐term impact (see Table 6).

**TABLE 6:** EIEQ item response distribution (*n*, %).

Item	True	Partly true	Neutral	Not true	Not applicable
The approach of using offering, guided interaction, and a supportive communication style has helped my child manage their eating difficulties.	14 (56.0%)	10 (40.0%)	1 (4.0%)	0 (0.0%)	0 (0.0%)
2 By practicing during meals under professional supervision, I have acquired useful tools to increase my child's interest in eating.	21 (84.0%)	4 (16.0%)	0 (0.0%)	0 (0.0%)	0 (0.0%)
3 Thanks to the continuity of the treatment, we have had a good opportunity to benefit from it.	22 (88.0%)	3 (12.0%)	0 (0.0%)	0 (0.0%)	0 (0.0%)
4 Meeting with a dedicated team of specialists throughout the treatment period has been valuable.	24 (96.0%)	1 (4.0%)	0 (0.0%)	0 (0.0%)	0 (0.0%)
5 It has been important for us as caregivers to discuss our child's eating difficulties – with one another and with specialists—without our child's presence.	24 (96.0%)	1 (4.0%)	0 (0.0%)	0 (0.0%)	0 (0.0%)
6 I have learned how play can be used to help my child engage with food.	23 (92.0%)	1 (4.0%)	1 (4.0%)	0 (0.0%)	0 (0.0%)
7 I have gained a better understanding of how sensory experiences affect my child's eating behavior.	20 (80.0%)	4 (16.0%)	1 (4.0%)	0 (0.0%)	0 (0.0%)
8 I have received expert support in assessing my child's nutritional, energy, and fluid needs.	24 (96.0%)	1 (4.0%)	0 (0.0%)	0 (0.0%)	0 (0.0%)
9 I have been supported in organizing our family's mealtime structure.	15 (60.0%)	5 (20.0%)	4 (16.0%)	0 (0.0%)	1 (4.0%)
10 I have observed an increased interest in eating in my child as a result of the intervention.	14 (58.3%)	4 (16.7%)	1 (4.2%)	5 (20.8%)	0 (0.0%)
11 I have observed improvements in my child's eating as a result of the intervention.	12 (50.0%)	5 (20.8%)	5 (20.8%)	1 (4.2%)	1 (4.2%)

*Note*: EIEQ = Eating Intervention Experience Questionnaire. Items were rated on a 7‐point Likert scale (1 = “Not true” to 7 = “Certainly true”), with an optional 0 = “Not applicable”. For this table, responses were recoded as follows: 7 = “Certainly true”, 5–6 = “Partly true”, 4 = “Neutral”, 1–3 = “Not true”, and 0 = “Not applicable”. Twenty‐five parents completed EIEQ items 1–9; 24 completed items 10–11. Percentages are calculated based on the number of responses for each item. The full 0–7 response distribution is presented in Table S1.

### 3.4 Qualitative Findings From Open‐Ended ESQ Responses

In addition to the quantitative results, parents' open‐ended responses offered further insight into their experiences of care. Thematic analysis identified three core themes.

#### 3.4.1 Skilled and Individualized Care

Parents consistently emphasized the value of care that was both personalized to their child's unique needs and delivered by knowledgeable, supportive staff. They described feeling genuinely understood, often for the first time in their child's care journey. As one parent reflected: “For the first time in my child's life, healthcare professionals took the time to really understand her challenges—and how to support her based on what she actually needs” (Parent 6). Parents also highlighted staff competence and their ability to engage children in new ways, as one described: “A complete stranger got our son to explore, touch, lick, taste – and even eat—new foods through play. That was incredible to witness” (Parent 25).

#### 3.4.2 Practical Support and Lasting Tools

Families valued the opportunity to try concrete strategies during the intervention and to take away tools they could continue using at home. Parents described receiving hands‐on coaching, personalized feedback, and useful ideas to support eating in daily life. For example: “You offered creative ideas and practical strategies we could try that really helped our child” (Parent 3). Another added: “We received hands‐on coaching and feedback, tried out activities we could continue at home, and got a thorough review of their nutrition needs” (Parent 28).

#### 3.4.3 Worries About Continuity of Support

While parents were positive about the care, many expressed worries about what would happen once the intervention ended. They feared losing access to specialized expertise and support, and some were anxious about maintaining progress over time. One parent explained: “It's worrying to think this kind of help won't be available anymore” (Parent 18). Another shared: “I'm worried about my daughter's future development without continued access to the kind of knowledge and support we received here” (Parent 8).

## 4 Discussion

This study evaluated clinical outcomes and parental experiences following an IMI for young children with ARFID at the FBHC, all of whom had longstanding, severe eating and feeding difficulties that had not improved despite first‐line treatment provided through local services. Although the vast majority of children continued to meet diagnostic criteria for ARFID at follow‐up, significant reductions in symptom severity (*d* = 0.80)—particularly within the sensory (*d* = 0.63) and low‐interest profiles (*d* = 0.93)—suggested meaningful clinical progress in this high‐need population. Given that ARFID often follows a long course (Lange et al. 2019; Zimmerman and Fisher 2017), these findings suggest that a brief, intensive approach may help initiate meaningful change—even if most children still met diagnostic criteria at follow‐up.

Beyond severe eating difficulties, most children presented with co‐occurring medical and/or developmental concerns and complex histories, consistent with those seen in pediatric feeding clinics. In this context, even partial improvements represent a realistic and valuable treatment outcome (Lenz et al. 2018; Sharp et al. 2016, 2020; Volkert et al. 2021). Parents' accounts reinforced this, with many describing even small steps as important—particularly in light of their child's persistent eating difficulties and limited response to previous treatment.

In addition to reductions in ARFID severity and high parental satisfaction, improvements were observed in specific diagnostic criteria. The proportion of children meeting criteria for weight loss/faltering growth (A1) and nutritional deficiency (A2) declined from 39% to 14% and from 18% to 7%, respectively. Although these criteria were not the most prevalent in our sample, the changes nonetheless represent meaningful clinical improvements, given the medical risks associated with malnutrition (James et al. 2024). By contrast, dependence on supplements or tube feeding (Criterion A3) and psychosocial impairment (A4) remained highly prevalent at follow‐up (A3: 82%; A4: 93%), underscoring ongoing functional challenges. It is plausible that the high rates of A3 in our sample helped mitigate the risk of A1 and A2, contributing to the relatively low prevalence of weight/growth and nutritional concerns across timepoints.

While this compensatory function may reduce immediate medical risk and provide reassurance to caregivers, it often introduces secondary difficulties—particularly in social contexts (Feillet et al. 2019; Krom et al. 2017; Taylor and Taylor 2021). For many children in our sample, nutritional dependence contributed directly to psychosocial impairment, including feelings of shame about consuming supplements in front of peers or requiring adult assistance during meals. These difficulties may intensify with age, as social awareness, sensitivity to peer comparison, and need for autonomy increase—potentially reinforcing avoidance and distress. Thus, strategies that address somatic risk may inadvertently sustain psychosocial difficulties by limiting engagement in developmentally meaningful eating experiences.

Consistent with this, parents reported lingering concerns about their child's future functioning, including worries about losing access to the specialized, coordinated support they considered important for maintaining progress. Caregiver education is considered a core component of effective ARFID interventions, with caregivers acting as the primary agents of strategy implementation in everyday life (Perez and Pesce 2025), and many parents in this study felt the intervention equipped them with helpful tools. Nonetheless, achieving independence from supplements or tube feeding may require sustained, structured support. Despite significant improvements at follow‐up, the persistence of A3 and A4 indicates a need for ongoing, coordinated care, including stepwise reduction of nutritional support, dietetic follow‐up, and targeted interventions to promote oral intake and functional independence (Feillet et al. 2019; Krom et al. 2017; Sharp et al. 2017; Taylor and Taylor 2021).

Building on previous IMI studies in similar populations—which have largely described directive behavioral approaches (Sharp et al. 2016, 2020; Volkert et al. 2021)—the present intervention relied on nondirective strategies that emphasize low‐pressure, attuned interactions. Although behavioral interventions show promise, they typically involve adult‐led prompting and reinforcement strategies that may not suit all families or presentations. Yet, direct comparisons to identify for whom and under what circumstances different IMI approaches are most appropriate remain challenging, due to variation in intervention duration and treatment goals, as well as limited characterization of ARFID presentations and inconsistent use of validated outcome measures.

For instance, Sharp et al. (2020) and Volkert et al. (2021) examined IMI programs grounded in behavioral frameworks but focused on samples meeting one ARFID criterion (nutritional deficiency, A2, or tube dependence, A3) without broader assessment of ARFID profiles. Although both studies reported positive outcomes—for example, Sharp et al. (2020) found that 72% of tube‐fed participants were weaned from tube feeding 12 months post‐intervention, compared with only 1 of 11 tube‐fed children (1/11; 9%) in our sample at follow‐up—their interventions were substantially longer (averaging over 35 days) and centered on specific, criterion‐related treatment goals.

By contrast, the present study assessed clinical characteristics comprehensively using the PARDI and implemented a brief (8‐day) intervention aimed at promoting positive, developmentally supportive eating experiences and strengthening parental capacity to support their child's eating development. As such, the clinical outcomes, complemented by quantitative and qualitative parental data, suggest that nondirective models may represent a viable alternative for severe ARFID in young children, particularly those with combined sensory sensitivity and low interest in eating profiles.

A further distinction relates to intervention delivery. Unlike most IMI programs for comparable populations—where psychologists or behavior analysts typically lead behavioral interventions and conduct parent training—the present intervention was delivered without these roles (Sharp et al. 2024; Willmott et al. 2024). As such, it may also hold relevance for settings with limited access to specialized behavioral expertise.

### 4.1 Strengths and Limitations

This was an open trial without a comparison group, limiting the ability to attribute observed changes specifically to the intervention; other influences, such as the natural passage of time, cannot be ruled out. Moreover, as follows: follow‐up questionnaires were not administered to parents, we cannot assess the durability of their perceived competence or the extent to which they continued to apply the strategies at home. However, given the long‐standing nature of the children's eating difficulties and previous unsuccessful treatment attempts, spontaneous remission appears unlikely. While causal inferences cannot be drawn, clinical improvements over time, together with parent‐reported gains in understanding and strategies, suggest that the intervention may have contributed to the changes observed.

The relatively small sample size (*n* = 28) may also limit generalisability. Nonetheless, given the clinical complexity of severe ARFID, the sample reflects a highly relevant group for evaluating this type of intervention. Prior research on IMI in similar populations has largely relied on retrospective designs, limited use of validated outcome measures, and sparse reporting on ARFID presentations (Sharp et al. 2016, 2020; Volkert et al. 2021; Willmott et al. 2024). In this context, the present study contributes complementary prospective data, using validated diagnostic interviews together with quantitative and qualitative parental reports, to a still‐emerging field.

The need for substantial parental involvement may also have influenced the sample, favoring families more likely to engage with and benefit from the intervention—potentially limiting applicability to those with fewer resources or lower readiness for change. At the same time, caregiver participation is central to most recommended approaches for young children with ARFID, making the sample representative of families most likely to engage with such interventions in clinical settings. Delivery within routine care additionally supports the external validity and practical relevance of the findings.

Another limitation is that one clinician involved in delivering the intervention also conducted outcome assessments, which may have introduced bias. Although structured tools were used, the lack of independent raters could have influenced the interpretation of symptom change. In addition, follow‐up assessments were conducted at variable intervals (7–15 months post‐intervention) due to organizational restructuring, which temporarily reduced scheduling capacity. All participants were nonetheless successfully followed up, and this variability is considered a minor methodological constraint.

Despite these limitations, the study has several strengths. Its prospective design provides longitudinal data on a clinically complex and understudied group of young children with ARFID, for whom structured intervention research remains limited. Validated outcome measures were complemented by both quantitative ratings and qualitative accounts of parents' experiences of the intervention, allowing for a more comprehensive view of clinical progress and family experiences. Finally, fidelity was supported through shared team routines, structured schedules, and video‐based review of mealtimes, helping to ensure consistent delivery of the intervention's core components.

## 5 Conclusions

This study examined clinical outcomes and parental experiences following an IMI for young children with ARFID, a clinical population for whom evidence‐based treatments remain scarce. Reductions in ARFID severity and diagnostic features, alongside positive parental reports, suggest that the intervention may support meaningful improvements in children with complex eating difficulties, particularly those with combined sensory sensitivity and low interest in food. While progress was evident, reflected in medium to large effect sizes in severity and profile scores, the vast majority of children still met diagnostic criteria for ARFID at follow‐up, indicating a continued need for support. Although the sample was relatively small, it included a clinically complex group with severe, longstanding difficulties and co‐occurring health problems—a population for whom effective treatment options are most urgently needed. To our knowledge, this is among the first prospective IMI studies focused on young children with ARFID and provides preliminary evidence for the potential value of structured, team‐based interventions grounded in nondirective, responsive strategies and active caregiver involvement. Further research with larger samples, comparison groups, and long‐term follow‐up is needed to strengthen the evidence base and inform development of sustainable, accessible models of care for this vulnerable population.

## Author Contributions

**Helena Holmäng**, **Katarzyna Brimo**, and **Lisa Dinkler:** conceptualization/design. **Helena Holmäng** and **Katarzyna Brimo:** data curation. **Helena Holmäng:** formal analysis. **Lisa Dinkler** and **Maria Råstam:** funding acquisition. **Anna Ås:** investigation. **Helena Holmäng**, **Katarzyna Brimo**, **Anna Ås**, and **Lisa Dinkler:** methodology. **Helena Holmäng**, **Katarzyna Brimo**, and **Lisa Dinkler:** project administration. **Helena Holmäng**, **Katarzyna Brimo**, and **Rachel Bryant‐Waugh:** resources. **Maria Råstam** and **Maj‐Britt Posserud:** supervision. **Helena Holmäng:** writing – original draft, visualization. All authors: writing – review and editing and approved submitted version.

## Funding

This work was supported by the Swedish Brain Foundation (Hjärnfonden) with support from Susanne Hobohms Stiftelse (Råstam, FO2020‐0140, FO2022‐0094). The funding bodies were not involved in the design of the study; collection, analysis, and interpretation of the data; or writing of the manuscript.

## Ethics Statement

The study was approved by the Swedish Ethical Review Authority (reference number: 2020‐01284, 2020‐03908). All participants gave written informed consent. All methods were conducted in compliance with the applicable guidelines and regulations.

## Use of Artificial Intelligence

Artificial intelligence tools were not used in the study design, data collection, analysis, or interpretation of results. AI‐based language tools were used during manuscript preparation to improve clarity and conciseness in the abstract and introduction only. All content was reviewed and approved by the authors, who take full responsibility for the manuscript.

## Involvement of Person with Lived Experience

Persons with lived experience were not involved in the study design or execution, or in the preparation of this manuscript.

## Conflicts of Interest

The authors declare no conflicts of interest.

## Supporting information


**Data S1:** eat70030‐sup‐0001‐Supinfo.docx.

## Data Availability

The data that support the findings of this study are available from the corresponding author upon reasonable request.
