# Supplementary material for: Intensive Multidisciplinary Intervention for Young Children With ARFID: Clinical Outcomes and Parental Experiences From a Prospective Cohort Study
Source: Int J Eat Disord. 2026 Jan 16;59(4):803–14. doi: 10.1002/eat.70030 (PMC13058405; doi:10.1002/eat.70030)
Supplement: Supplementary file 1 — Data S1: eat70030‐sup‐0001‐Supinfo.docx. [file EAT-59-803-s001.docx]

**Supplementary Summary 1**

*Core Principles of the Guidance during Meals Approach.* This approach, developed in clinical practice at the Folke Bernadotte Regional Habilitation Center (FBHC) in Uppsala, Sweden, is grounded in non-directive, responsive interaction strategies aimed at supporting children with eating difficulties. It is structured around two core principles: Offer and Guide.

**Offer**

The foundation of offering is to avoid any form of pressure – including coaxing, persuading or “tricking” the child. Food should not be physically brought close unless the child signals readiness. The child’s comfort zone must be respected. Trust is built by waiting for subtle cues of willingness to engage, such as leaning forward, looking, smelling, licking or tasting.

- Use a positive and accepting tone. The child’s reactions – whether hesitant or exploratory – should be acknowledged and respected.
- Observe the child’s cues carefully. If signs of distress, fatigue or disengagement appear, the interaction should be paused or stopped.
- Avoid questions about eating, as they often invite refusal and place unnecessary cognitive demands on the child. Instead of asking, name what is happening (see *Guide* below).
- Avoid commands, which can create a sense of pressure. Guidance is offered through modelling and descriptive language.
- Avoid praise related to eating. Instead, affirm the child’s efforts through neutral descriptions of their actions. This helps to avoid creating performance pressure or interpreting missed attempts as failure.

**Guide**

Guidance involves structuring the mealtime experience in a predictable and supportive way.

- Establish a clear beginning and end to meals using simple rituals (e.g., handwashing, saying “thank you” as a cue to end the meal – not as a moral lesson).
- Use narration and naming throughout the meal. For example:

“I’m slicing some cheese.”
“You’re tasting now.”
“You can try using the spoon.”
This fosters engagement, clarity and a sense of agency.

- Model actions by demonstrating possibilities: taste, smell or explore the food yourself.
- Mirror the child’s actions – show that you are attuned and inspired by their exploration.
- Use turn-taking to create rhythm and shared interaction:
  1. “Now Mum smells the cheese. Mmm, that smells good.”
  2. “Now Kalle can smell it – oh, you’re looking at the cheese.”
  3. “Now I’ll smell it again.”

This approach supports the child’s autonomy while encouraging participation.

- Foster positive associations with food through expressive facial cues and tone of voice. A pleasant mealtime atmosphere can motivate the child to re-engage in future meals.
- Engage all senses – sight, hearing, smell, touch (hands, face, mouth) and taste – at the child’s own pace.
- Use imagination and playfulness where appropriate. Siblings can help explore food. Wait *with* the child, but do not *wait out* the child in order to follow an adult-led agenda.

**Supplementary Form 1**

*Eating Intervention Experience Questionnaire (EIEQ)*. The questionnaire consists of 11 items rated on a 7-point Likert scale (1 = Not True to 7 = Certainly True), with an optional “Not Applicable” response coded as 0.

|  | Certainly True |  |  | Neither agree nor disagree |  |  | Not True | Not Applicable |
| --- | --- | --- | --- | --- | --- | --- | --- | --- |
| 1. The approach of using offering, guided interaction, and a supportive communication style has helped my child manage their eating difficulties. | 7 | 6 | 5 | 4 | 3 | 2 | 1 | 0 |
| 2. By practicing during meals under professional supervision, I have acquired useful tools to increase my child's interest in eating. | 7 | 6 | 5 | 4 | 3 | 2 | 1 | 0 |
| 3. Thanks to the continuity of the treatment, we have had a good opportunity to benefit from it. | 7 | 6 | 5 | 4 | 3 | 2 | 1 | 0 |
| 4. Meeting with a team of specialists throughout the treatment period has been valuable. | 7 | 6 | 5 | 4 | 3 | 2 | 1 | 0 |
| 5. It has been important for us as caregivers to discuss our child's eating difficulties – with one another and with specialists – without our child's presence. | 7 | 6 | 5 | 4 | 3 | 2 | 1 | 0 |
| 6. I have learned how play can be used to help my child engage with food. | 7 | 6 | 5 | 4 | 3 | 2 | 1 | 0 |
| 7. I have gained a better understanding of how sensory experiences affect my child’s eating behaviour. | 7 | 6 | 5 | 4 | 3 | 2 | 1 | 0 |
| 8.  I have received expert support in assessing my child's nutritional, energy, and fluid needs. | 7 | 6 | 5 | 4 | 3 | 2 | 1 | 0 |
| 9. I have been supported in organizing our family's mealtime structure. | 7 | 6 | 5 | 4 | 3 | 2 | 1 | 0 |
| 10. I have observed an increased interest in eating in my child as a result of the treatment. | 7 | 6 | 5 | 4 | 3 | 2 | 1 | 0 |
| 11. I have observed improvements in my child's eating as a result of the treatment. | 7 | 6 | 5 | 4 | 3 | 2 | 1 | 0 |

**Supplementary Table 1**

*Distribution of responses on the EIEQ (n, %).* A total of 25 parents (89.3%) completed the questionnaire; two responses were missing for items 10–11 (n = 24).

| Item | 7  Certainly true | 6 | 5 | 4  Neither agree nor disagree | 3 | 2 | 1  Not true | 0  Not applicable |
| --- | --- | --- | --- | --- | --- | --- | --- | --- |
| 1. The approach of using offering, guided interaction, and a supportive communication style has helped my child manage their eating difficulties. | 14 (56.0 %) | 9 (36.0 %) | 1 (4.0%) | 1 (4.0%) | 0 | 0 | 0 (0.0 %) | 0 (0.0 %) |
| 2. By practicing during meals under professional supervision, I have acquired useful tools to increase my child's interest in eating. | 21 (84.0 %) | 2 (8.0 %) | 2 (8.0 %) | 0 (0.0 %) | 0 (0.0 %) | 0 (0.0 %) | 0 (0.0 %) | 0 (0.0 %) |
| 3. Thanks to the continuity of the treatment, we have had a good opportunity to benefit from it. | 22 (88.0 %) | 3 (12.0 %) | 0 (0.0 %) | 0 (0.0 %) | 0 (0.0 %) | 0 (0.0 %) | 0 (0.0 %) | 0 (0.0 %) |
| 4. Meeting with a dedicated team of specialists throughout the treatment period has been valuable. | 24 (96.0 %) | 0 (0.0 %) | 1 (4.0 %) | 0 (0.0 %) | 0 (0.0 %) | 0 (0.0 %) | 0 (0.0 %) | 0 (0.0 %) |
| 5. It has been important for us as caregivers to discuss our child's eating difficulties – with one another and with specialists – without our child's presence. | 24 (96.0 %) | 0 (0.0 %) | 1 (4.0 %) | 0 (0.0 %) | 0 (0.0 %) | 0 (0.0 %) | 0 (0.0 %) | 0 (0.0 %) |
| 6. I have learned how play can be used to help my child engage with food. | 23 (92.0 %) | 1 (4.0 %) | 0 (0.0 %) | 1 (4.0 %) | 0 (0.0 %) | 0 (0.0 %) | 0 (0.0 %) | 0 (0.0 %) |
| 7. I have gained a better understanding of how sensory experiences affect my child’s eating behaviour. | 20 (80.0 %) | 4 (16.0 %) | 0 (0.0 %) | 1 (4.0 %) | 0 (0.0 %) | 0 (0.0 %) | 0 (0.0 %) | 0 (0.0 %) |
| 8. I have received expert support in assessing my child's nutritional, energy, and fluid needs. | 24 (96.0 %) | 1 (4.0 %) | 0 (0.0 %) | 0 (0.0 %) | 0 (0.0 %) | 0 (0.0 %) | 0 (0.0 %) | 0 (0.0 %) |
| 9. I have been supported in organizing our family's mealtime structure. | 15 (60.0 %) | 5 (20.0 %) | 0 (0.0 %) | 4 (16.0 %) | 0 (0.0 %) | 0 (0.0 %) | 0 (0.0 %) | 1 (4.0 %) |
| 10. I have observed an increased interest in eating in my child as a result of the treatment. | 14 (58.3 %) | 4 (16.7 %) | 0 (0.0 %) | 1 (4.2 %) | 4 (16.7 %) | 1 (4.2 %) | 0 (0.0 %) | 0 (0.0 %) |
| 11. I have observed improvements in my child's eating as a result of the treatment. | 12 (50.0 %) | 1 (4.2 %) | 4 (16.7 %) | 5 (20.8 %) | 0 (0.0 %) | 0 (0.0 %) | 1 (4.2 %) | 1 (4.2 %) |
